# Tapetum-specific expression of a cytoplasmic *orf507* gene causes semi-male sterility in transgenic peppers

**DOI:** 10.3389/fpls.2015.00272

**Published:** 2015-04-22

**Authors:** Jiao-Jiao Ji, Wei Huang, Zheng Li, Wei-Guo Chai, Yan-Xu Yin, Da-Wei Li, Zhen-Hui Gong

**Affiliations:** ^1^College of Horticulture, Northwest A&F UniversityYangling, China; ^2^State Key Laboratory of Stress Biology for Arid Areas, Northwest A&F UniversityYangling, China; ^3^Institute of Vegetables, Hangzhou Academy of Agricultural SciencesHangzhou, China

**Keywords:** *Capsicum annuum* L., *orf507*, cytochrome *c* oxidase, tapetum, transgenic semi-male sterility

## Abstract

Though cytoplasmic male sterility (CMS) in peppers is associated with the *orf507* gene, definitive and direct evidence that it directly causes male sterility is still lacking. In this study, differences in histochemical localization of anther cytochrome *c* oxidase between the pepper CMS line and maintainer line were observed mainly in the tapetal cells and tapetal membrane. Inducible and specific expression of the *orf507* gene in the pepper maintainer line found that transformants were morphologically similar to untransformed and transformed control plants, but had shrunken anthers that showed little dehiscence and fewer pollen grains with lower germination rate and higher naturally damaged rate. These characters were different from those of CMS line which does not produce any pollen grains. Meanwhile a pollination test using transformants as the male parent set few fruit and there were few seeds in the limited number of fruits. At the tetrad stage, ablation of the tapetal cell induced by premature programmed cell death (PCD) occurred in the transformants and the microspores were distorted and degraded at the mononuclear stage. Stable transmission of induced semi-male sterility was confirmed by a test cross. In addition, expression of *orf507* in the maintainer lines seemed to inhibit expression of *atp6-2* to a certain extent, and lead to the increase of the activity of cytochrome *c* oxidase and the ATP hydrolysis of the mitochondrial F_1_F_o_-ATP synthase. These results introduce the premature PCD caused by *orf507* gene in tapetal cells and semi-male sterility, but not complete male sterility.

## Introduction

Cytoplasmic male sterility (CMS) is a maternally inherited trait that prevents a plant from producing functional pollen grains. CMS has been used widely to produce F_1_hybrids in order to increase fruit yield and decrease the energetic (Budar and Pelletier, 2001). The economically viable production of hybrid seeds requires a good pollination control system to avoid self-pollination of the female line. The CMS lines, that can-not produce functional pollen grains, are often used as female lines in hybrid seed production, to increase the purity of seeds (Schnable and Wise, 1998). However, the several bottlenecks faced during the development of CMS lines through conventional breeding require biotechnological intervention. The plant mitochondrial genome cannot be manipulated directly, so investigations of mitochondrial contributions to male sterility must be made by engineering nuclear genes encoding mitochondria-targeted proteins, including the expression of unedited forms of mitochondrial genes in the maintainer line (Hernould and Suharsono, 1993), and the expression of the CMS-associated mitochondrial *orf*s (Yamamoto et al., 2008). In most cases, CMS was related with premature degradation of the tapetal cell, a sporogenous tissue that nurtures the pollen mother cells (Hernould et al., 1998). In sunflowers, the CMS-associated open reading frame (ORF), *orf*H522, induced male sterility after being introduced into tobacco plants and expressed under the tapetum-specific promoter TA29. Bright-field microscopy demonstrated ablation of the tapetal cell layer in transgenic sterile plants that contain the *orf*H522 gene. Premature DNA fragmentation and programmed cell death (PCD) were observed at meiosis stage in the anthers of sterile plants (Nizampatnam et al., 2009).

It is generally believed that CMS results from the rearrangement of mitochondrial genomes, which is attributed to the generation of novel *orfs* (Schnable and Wise, 1998; Budar and Pelletier, 2001; Hanson and Bentolila, 2004; Linke and Börner, 2005; Yang and Zhang, 2007). Some experimental evidence has confirmed the correlation between CMS-associated *orfs* and the occurrence of CMS (Hanson and Bentolila, 2004). In *Brassica juncea*, transgenic plants that contained the CMS-associated *orf220* gene and a mitochondrial-targeting sequence of the β subunit of F_1_-ATPase were male-sterile. In addition, transgenic stem mustard plants had aberrant floral development, identical to what has been observed in the CMS stem mustard phenotype (Yang et al., 2010). As described above, targeted expression in mitochondria of novel *orfs* has been shown to lead to male sterility or semi-sterility in some cases (He et al., 1996; Wang et al., 2006; Kim et al., 2007; Yamamoto et al., 2008), while in other cases, there was no effect (Chaumont et al., 1995; Wintz et al., 1995; Duroc et al., 2006). Most of the *orf*s in CMS lines are related to the mitochondrial energy metabolism complex. For example, *orf522* in sunflower PET1-CMS encodes a protein sharing 18 amino acids with ORFB which is homologous to ATP8 in *Reclinomonas* (Balk and Leaver, 2001), and *orf79* in rice is co-transcribed with the *B-atp6* gene and encodes a cytotoxic peptide (Wang et al., 2006). All novel *orfs* have been confirmed to be associated with CMS. In addition, mutation of genes encoding subunits of mitochondrial enzymes also induces male sterility. For instance, mutation in the *MGP1* gene, encoding the F_A_d subunit of F_1_F_o_-ATP synthase in *Arabidopsis thaliana*, led to the destruction of mitochondria in pollen grains and subsequently the death of the pollen grains (Li et al., 2010).

In pepper (*Capsicum annuum* L.), CMS was first isolated from an Indian *C. annuum* accession (PI164835) (Peterson, 1958). Molecular investigations revealed that male sterility in many CMS lines is associated with the expression of *orf456*, which is located downstream and co-transcribed with the mitochondrial *coxII* gene (Kim et al., 2007), and the pseudogene *ψatp6-2*, 3′-truncated form of *atp6-2* in maintainer line (Kim and Kim, 2006). Expression of the mitochondrion-targeted *orf456* gene under the tapetum-specific promoter TA29 in transgenic *Arabidopsis* has shown that 45% of the T_1_ transgenic population is male-sterile and had no seed set, and the pollen grains of semi-sterile T_1_ plants have exine layer defects and vacuolated pollen phenotypes (Kim et al., 2007). However, an altered transcript, *orf507*, was found in a sequencing error at the 3′ end of *orf456* (Gulyas et al., 2010). Expression of the *orf507* gene in the flowers and leaves encodes a toxic protein that inhibits cell growth, and specifically interacts with a subunit of ATP synthase 6 (Li et al., 2013). In CMS plants, ATP synthase activity and content was reduced by more than half, compared normal plants. Based on these results, the researchers speculated that ORF507 might cause MtATP6 to be nonfunctional (Li et al., 2013). However, empirical proof that ORF507 is sufficient to induce male sterility has not been provided. In a previous study, we found that mitochondrial cytochrome *c* oxidase and F_1_F_o_-ATPase were dysfunctional in the CMS line HW203A (Ji et al., 2013), but it remains unclear whether expression of *orf507* is responsible for the dysfunction of the mitochondrial enzyme.

Therefore, we hypothesized that if *orf507* is expressed under the tapetum-specific promoter TA29 from tobacco, and the protein is targeted into mitochondria using the *coxIV* pre-sequence as the transit peptide, ORF507 protein introduced into maintainer lines might result in male sterility and dysfunction of the mitochondrial enzyme in pepper plants. The *coxIV* pre-sequence have been proved to target the in-frame proteins into mitochondria in heterologous systems without any error into the chloroplast (Schmitz and Lonsdale, 1989). Kim et al. (2007) demonstrated that the *coxIV* pre-sequence was successfully used for targeting ORF456 from pepper into mitochondria in a heterologous plant. Simultaneously, we analyzed the histochemical localization of cytochrome *c* oxidase in anthers of different developmental stages in the pepper CMS line and maintainer line to evaluate the relationship between cytochrome *c* oxidase and male sterility.

## Materials and methods

### Plant materials and growth conditions

The pepper (*Capsicum annuum* L.) CMS line HW203A and its near-isogenic maintainer line HW203B (WT) were provided by the Asian Vegetable Research and Development Center (AVRDC). The CMS line was used as non-recurrent parent and maintainer line (WT), as recurrent parent. Eight generations of back-crosses were developed to obtain a CMS line similar to a maintainer line with CMS transferred from non-recurrent parents (CMS line). After eight backcrosses, the developed line (CMS line) is an isogenic line with it's maintainer line (WT), but the cytoplasmic background was different in the CMS and WT lines.

*Capsicum annuum* used in this study were grown in plastic trays containing steam-sterilized growing medium at 25°C with a 16 h light/8 h dark cycle.

### Histochemical localization of cytochrome c oxidase in anthers at different developmental stages

Anthers were collected from the CMS line (HW203A) and the maintainer line (HW203B) at the microspore mother cell stage (MMC), tetrad stage and mononuclear microspore (MNM) stage at 4°C to avoid the inactivity of the enzyme. At each stage of anther development, five anthers were randomly selected from five pepper plants. The anthers were soaked in a fixation solution (2.5% glutaraldehyde, 1%TritonX-100) for 20 min at 4°C, washed with 0.1 M PBS (pH 7.4), and then incubated in a PBS buffer solution containing 0.1 mg/mL catalase for 10 min. The anthers were washed again in PBS three times, and then incubated in a 0.1 M PBS buffer solution containing 1, 0.1, and 1 mg/mL cyt *c* for 1 h. 1 mM KCN was added in the control. Sample fixation and observation was conducted following the protocols outlined in Ji et al. (2014a).

### Vector construction

To construct the vector, a tapetum-specific promoter (TA29) from tobacco and a mitochondrial transit sequence (the first 25 amino acids of COXIV) from yeast were cloned. The *orf507* gene was taken from the HW203A CMS line of *C. annuum*. Primers used in this study (Table 1) were designed for amplification of the component sequences from their respective sources, and restriction enzyme sites were introduced at the 5′ end of the primers. The PCR amplified component sequences were first cloned in the pMD19-T vector, sequenced, and excised with specific enzymes that were introduced into the primers. The TA29 promoter, *coxIV* pre-sequence and *orf507* were cloned sequentially into the pCAMBIA 2300 vector. The resultant clone pTCON (pCAMBIA 2300: TA29-*coxIV* pre-sequence—*orf507*—nos terminator) was used for plant transformation. The control was constructed without the objective gene *orf507*, pTCN (pCAMBIA 2300: TA29-*coxIV* pre-sequence—nos terminator). The pSCON vector (pCAMBIA 2300: CaMV35S-*coxIV* pre-sequence—*orf507*—nos terminator) was also constructed for the comparison with the pTCON transgenic plants. The clones were confirmed by restriction analysis, PCR and sequencing. The confirmed clones were then transformed into *Agrobacterium tumefaciens* strain GV3101, following the standard freeze thaw method of transformation (Ji et al., 2014a).

**Table 1:** **List of primers used in this study**.

**Primer code**	**Sequence (5′–3′)**	**Purpose of primers**
TA29F	5′-ACACAAATAGCCGGCTATAT-3′	Amplification of TA29 promoter
TA29R	5′-GCTTCGAATAGCTAATTTCTTTAAGTAAAAAC-3′	
*Bam*H I *coxIV*F	5′-CGCGGATCCATGCTTTCACTACGTCAATCTATAAG-3′	Amplification of the transit peptide
*Kpn* I *coxIV*R	5′-CGGGGTACCACCCTCTTTAGCACCAGGACC-3′	
*Kpn* I *orf507*F	5′-CGGGGTACCATGCCCAAAAGTCCCATGT-3′	Amplification of *orf507* gene
*Sac* I *orf507*R	5′-CGAGCTCTTAAAAAGCGCTAAACAAAT-3′	
*caubi* F	5′-TGTCCATCTGCTCTCTGTTG-3′	qRT-PCR
*caubi* R	5′-CCCAAGCACAATAAGAC-3′	qRT-PCR
q*coxII* F	5′-ATCCAGCCATTACTATCAAA-3′	qRT-PCR
q*coxII* R	5′-GTACAGCCCAACTATCAGG-3′	qRT-PCR
q*507*F	5′-ATGCCCAAAAGTCCCATGT-3′	qRT-PCR
q*507*R	5′-AAAAGCGCTAAACAAATTGC-3′	qRT-PCR
r *atp6-2*F	5′-GCTTCGCCTTCGTATAGTAGTTC-3′	qRT-PCR
r *atp6-2*R	5′-AGTCATAGTGCTCACCCTGTTTG-3′	qRT-PCR

*The restriction enzyme sites added at 5′ end of primers are underlined*.

### Plant transformation

The constructed TCON was used to transform the maintainer line of pepper (HW203B), via leaf disk transformation procedure. Sterile explants were infected with the GV3101 strain (OD600 = 0.5~0.8) containing TCON for 10 min. The infected explants were co-cultured in a solid MB medium for 3 d until the single colony had formed, and then transferred into another medium (MB + 50 mg/L Cef). When adventitious buds began to grow, the explants were cultured in a medium (MB + 50 mg/L Kan + 50 mg/L Cef). Induction, differentiation and elongation of the adventitious buds were executed according to procedures outlined by Wang et al. (Wang, 2007). Finally, the elongated adventitious buds were cultured in a solid medium containing 1/2 MS + 50 mg/L Kan + 50 mg/L Cef (pH 5.8) for root production. The plants were acclimatized initially in a tissue culture room and later transferred to an illumination incubator. The transformed control plants were infected with the *Agrobacterium* strain containing TCN. The T_0_ male sterile plants obtained were pollinated with the pollen from transformed control pepper plants to obtain BC_1_T_0_ generation seeds.

### Selection and detection of the transformed pepper

Genomic DNA was isolated from the leaves of transformed and transformed control plants using the CTAB method to check for transgenicity (Ji et al., 2014b). PCR analysis using *Bam*HI *coxIV*F and *Sac*I *orf507*R primers was performed to confirm the presence of the gene cassette in the transgenic plants. Plants that were positive for gene-specific PCR were subjected to RT-PCR (reverse transcription PCR) and qRT-PCR (quantitative real time PCR) analysis using cDNA from flower buds at the tetrad stage, leaves as the template, and the *ubiquitin-conjugating protein* gene (*caubi* F, *caubi* R) as the reference gene. The cDNA of transformed control plants was used as the negative control, with the *coxII* gene as the reference gene. RNA isolation of the flower buds and leaves was conducted according to Ji et al. (2013). The two sets of primers used for RT-PCR and qRT-PCR analysis were *Kpn* I *orf507*F, *Sac*I *orf507*R and q*507*F, q*507*R, respectively (sequences listed in Table 1). The TCON vector was used as the positive control.

### Pollen analysis

After flowering, transgenic and control plants were scored for pollen abundance, pollen characteristics and germination. Non-dehisced anthers from 10 flowers selected randomly from different plants were collected into 2 mL tubes containing 400 μL 1% sodium metaphosphate and placed in a dryer to release the pollen grains. Natural damage rate of the pollen grains was then observed. Fresh pollen grains from five flowers collected from different plants were mixed randomly to assess the germination rate according to the protocol described by Ji et al. (2014a). Pollen viability was tested with the acetocarmine staining method (Malayeri et al., 2012). The nucleus of the viable pollen grains can be stained deep red, while that of the non-viable pollen grains cannot be stained. The CMS lines and the transformed control plants were used as the controls. All the experiments were replicated three times.

Based on the rate of pollen damage and germination results, plants were classified as sterile, semi-sterile, and fertile. If no pollen grains were released and the anthers were wizened and browning, such plants were classified as completely male-sterile. If the anthers were wizened, more than 30% of pollen grains were damaged and less than 5% of pollen grains germinated, then such plants were classified as semi-sterile, while plants were designated as fertile if greater than 40% pollen grains were able to germinate.

### Light microscopy

At the mother microspore cell stage, tetrad stage and MNM stage, anthers were fixed in glutaraldehyde, dehydrated in an ethanol series, and embedded in Epon812 resin (Ji et al., 2014a). Using an automatic microtome (ULTRACUT, Germany) sections of 1 μm were then cut. A bright-field photograph of each anther cross-section was taken using a microscope.

### Pollination test

The untransformed pepper plants (female parent) were crossed with transgenic plants (male parent). Fifteen flowers were pollinated in each combination. Then the fruit-set and seeds in each fruit were counted. The transformed control pepper plant was used as male parent for the control.

### Progeny analysis

Transgenic plants (female parent) were crossed with untransformed plants (male parent) to obtain BC_1_T_0_ plants. The seeds obtained were subjected to a germination test on filter paper containing 150 mg/L *Kanamcin*. Seeds were scored as positively germinating if the radicles could grow normally and root hairs were present, and they were scored as negatively germinating if the radicles browned and died. The transgenic line with 1:1 ratio of positive:negative seeds was chosen as the material for molecular analysis of the test cross progeny.

Seeds of the selected transgenic line were cultured in plastic trays containing steam-sterilized growing medium at 25°C on a cycle of 16 h light/8 h dark. Gene-specific PCR analysis of the leaves was performed with *Bam*HI *coxIV*F and *Sac*I *orf507*R primers. RT-PCR detection of the BC_1_T_0_ progeny was similar to that for the T_0_ generation plants, using the primers *Kpn* I *orf507*F and *Sac*I *orf507*R. The 80 randomly selected progeny were used in the PCR.

### Expression pattern of the *orf507* and *atp6-2* gene in BC_1_T_0_ progeny

To assess interactions between the *orf507* and *atp6-2* genes, expression patterns of the two genes in BC_1_T_0_, CMS line and maintainer line were analyzed with qRT-PCR. RNA was isolated from anthers at the microspore mother cell (MMC) stage, tetrad stage and MNM stage, and transcribed to the cDNA template (Ji et al., 2013). Two sets of primers (q*507*F, q*507*R and r *atp6-2*F, r *atp6-2*R) were used to analyze the expression patterns of the *orf507* and *atp6-2* genes, respectively. In BC_1_T_0_, *ubiquitin-conjugating protein* gene was used as the reference gene for the analysis of the *orf507* gene and *coxII* gene was used as the reference gene for *atp6-2*, while for the analysis of the *orf507* and *atp6-2* genes in the CMS line and maintainer line, mitochondrial *coxII* was used as the reference gene. All of the sequences of the primers were listed in Table 1. All experiments were replicated three times.

### Activity of mitochondrial cytochrome c oxidase and ATP hydrolysis of F_1_F_o_-ATP synthase

At the tetrad stage in the BC_1_T_0_ progeny, mitochondria were isolated from anthers to measure the activity of mitochondrial cytochrome *c* oxidase and the ATP hydrolysis of F_1_F_o_-ATP synthase following established protocols (Ji et al., 2014a), using untransformed plants and the CMS line as controls. All the experiments were replicated three times.

## Results

### Histochemical localization of cytochrome c oxidase in anthers

**Figure 1:**
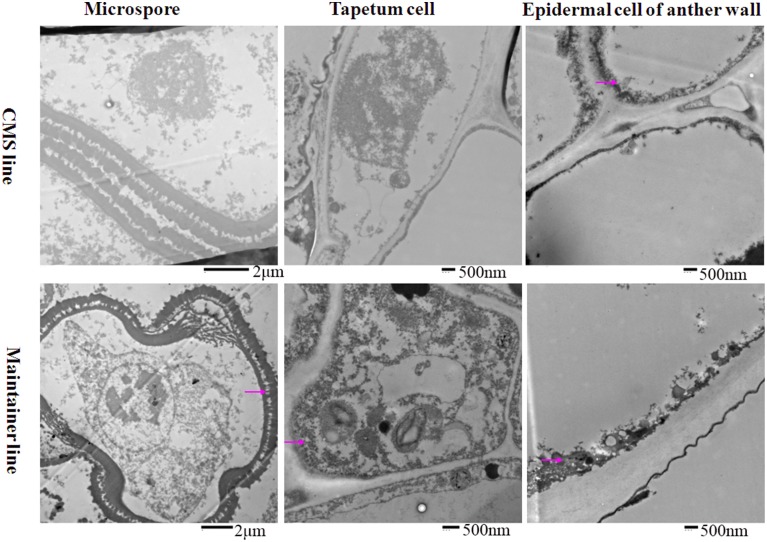
**Localization of cytochrome *c* oxidase in anthers at the MMC stage**. Purple arrow indicated black particles of cyt *c* oxidase reaction.

To analyze the relationship between cytochrome *c* (cyt *c*) oxidase and CMS, we performed histochemical localization of the enzyme in anthers of the CMS line (HW203A) and the maintainer line (HW203B). Reduction of oxidized cyt *c* by DAB leads to accumulation of DAB oxide that can react with the O_s_O_4_ to form osmium black, which can be viewed as black particles under TEM (Noda et al., 1994). The results showed that osmium black was localized on the membrane of the anther wall at the MMC stage in the CMS line, while hardly any osmium black particles were found in the microspore mother cell and the tapetum cell (Figure 1). All of these findings indicated that in the CMS line, cytochrome *c* oxidase was less active at the MMC stage than in the maintainer line. However, in the maintainer line osmium black particles were observed in the cytoplasm and nucleus of the microspore mother cell, the tapetum cell, and the membrane of the anther wall (Figure 1).

**Figure 2:**
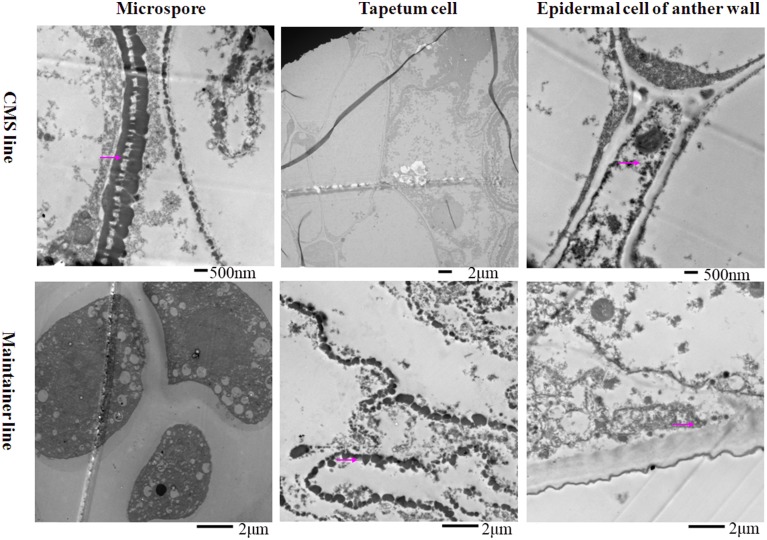
**Localization of cytochrome *c* oxidase in anthers at the tetrad stage**. Purple arrow indicated black particles of cyt *c* oxidase reaction.

At the tetrad stage, in the CMS line in which the tapetal cells started to degrade, the activity of the enzyme was concentrated mainly in the membrane and intercellular space of the anther wall, the cytoplasm and the space between the exine and intine of the tetrad microspores (Figure 2). In contrast, osmium black particles were uniformly distributed in the cells of the anther wall in the maintainer line, and numerous osmium black particles were concentrated in the tapetal cell and tapetal membrane (Figure 2). Few osmium black particles were observed in the tetrad microspores (Figure 2).

**Figure 3:**
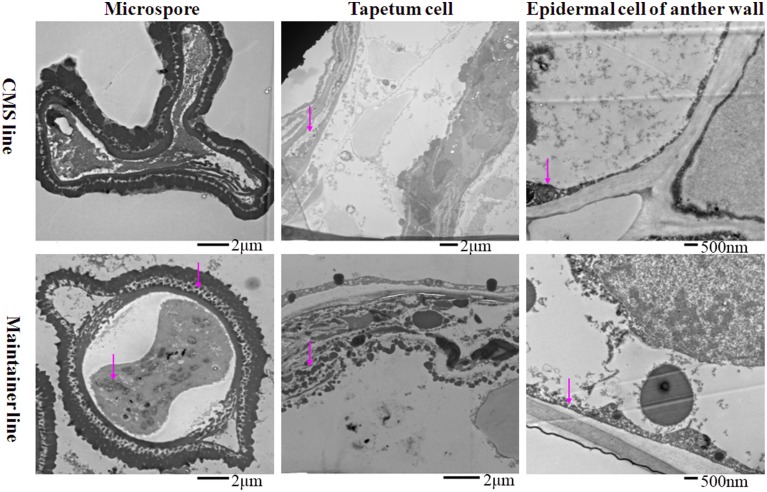
**Localization of cytochrome *c* oxidase in anthers at the MNM stage**. Purple arrow indicated black particles of cyt *c* oxidase reaction.

At the MNM stage in the CMS line, concentrated osmium particles were found in the membrane of the anther wall (Figure 3). The tapetal cells of the CMS line were degraded, and few osmium black particles were observed in the residuum (Figure 3). Meanwhile, the microspores were distorted (Figure 3). In the maintainer line, a strong enzymatic reaction occurred in the MNMs, the tapetal membrane and the residuum of the tapetum, especially in the cytoplasm and the space between the extine and intine of the MNMs (Figure 3). In addition, few osmium black particles were observed in the membrane and the nucleus of the anther wall (Figure 3).

All of these findings indicated that throughout the development of anthers, the reaction in the anthers of the enzyme cytochrome *c* oxidase showed significant differences between the CMS line and the maintainer line, especially in the tapetum after the tetrad stage. This indirect evidence might indicate the dysfunction of cytochrome *c* oxidase in the CMS line, combing with a former study that demonstrated that the changing trend of cytochrome *c* oxidase at different pollen development stages in the CMS line was contrary to that in the maintainer line (Ji et al., 2013).

### Transformation of pepper plants

**Figure 4:**
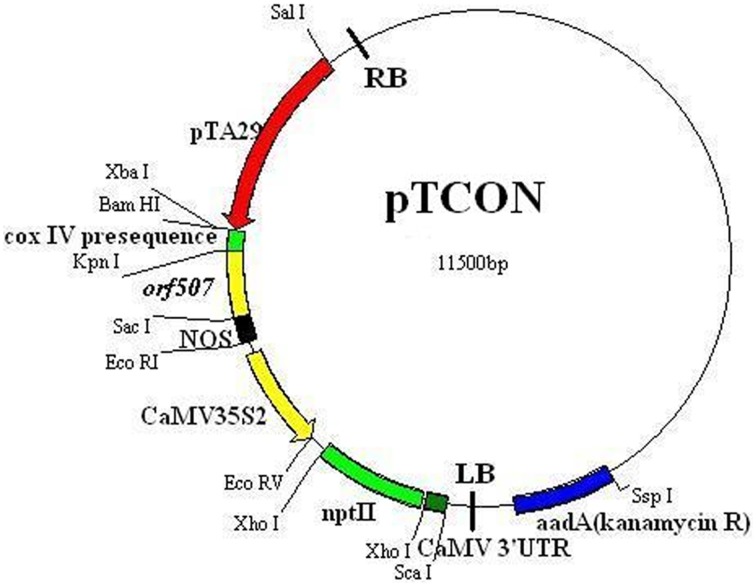
**Schematic illustration of pTCON vector construct**.

Analysis of the potential role of the *orf507* gene in causing the dysfunction of cytochrome *c* oxidase and *C. annuum* CMS was performed via the construction of the TCON vector using a *pCAMBIA2300* vector. Cytological observation of anther development showed premature PCD in the tapetum of the CMS line (HW203A), and significant differences in histochemical localization of cytochrome *c* oxidase also occurred in the anther tapetum. In this study, *orf507* was expressed under a tapetum-specific TA29 promoter, and then the expression products were transferred into the mitochondria via the *coxIV* pre-sequence that consisted of the first 25 amino acids of the *coxIV* gene isolated from yeast. The schematic illustration of the gene construct was shown in Figure 4.

**Figure 5:**
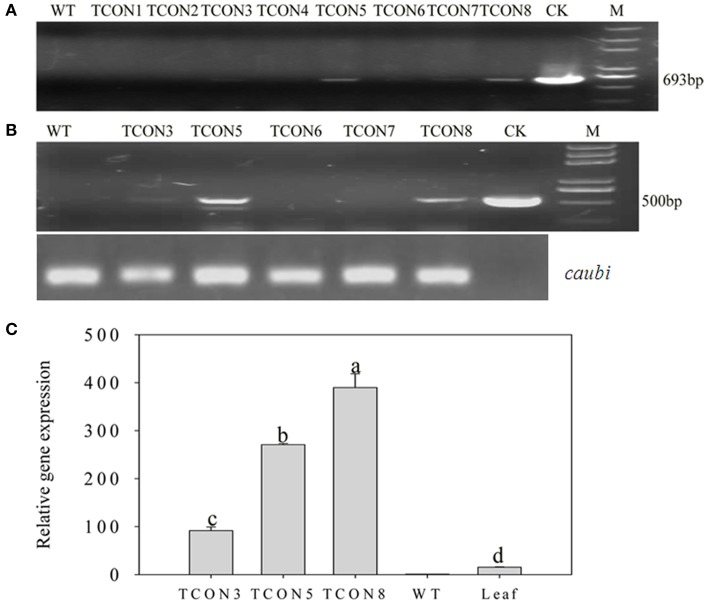
**Molecular analysis of T_0_ transgenic plants**. **(A)** PCR confirmation of TCON transgenic pepper plants using *Bam*HI *coxIV*F and *Sac*I *orf507*R primers that would amplify 693 bp fragment of the cassette in DNA level.**(B)** RT-PCR analysis of buds from TCON transgenic plants with *Kpn* I *orf507*F and *Sac*I *orf507*R primers that would amplify 507 bp fragment with *caubi* gene as reference gene. **(C)** qRT-PCR analysis of buds from TCON transgenic plants. TCON1~8, transgenic TCON plants; WT, transformed control plant; CK, positive control (amplified from plasmid DNA); Leaf, leaves collected from the three transformants; M, Trans2K plus marker. Statistically significant differences between the means were determined using Fisher's LSD test (*p* < 0.05).

Using an improved genetic transformation system (Wang, 2007), transformed plants were obtained throughout the shoot differentiation, elongation, and rooting stages (Supplementary Figure 1). There were no phenotypic abnormalities between the transformants and the controls that had been infected with the TCN vector without the *orf507* gene. The transformants were checked for the transgenicity using gene-specific PCR primers with the pTCON plasmid as the positive control and the non-transgenic plant as the negative control. Among the eight transformants obtained from explantation, five lines (TCON3, TCON5, TCON6, TCON7, and TCON8) showed the presence of the gene casette, while no PCR products were observed in the non-transgenic plants (WT) (Figure 5A).

Inspection for the presence of *orf507* transcripts in the anthers of PCR positive progeny plants revealed that only three transformants (TCON3, TCON5, and TCON8) were able to amplify the objective fragment. No fragments were amplified in the WT (Figure 5B). Further, qRT-PCR analysis showed that expression of the *orf507* gene was restricted to the buds and was basically not detected in the leaves of transgenic plants (Figure 5C), which indicated the anther-specific expression of *orf507*. Meanwhile the gene expression in TCON8 was significantly higher than that in TCON3 and TCON5, with no expression detected in the WT (Figure 5C).

### Morphological and cytological analysis of pollen grains

**Figure 6:**
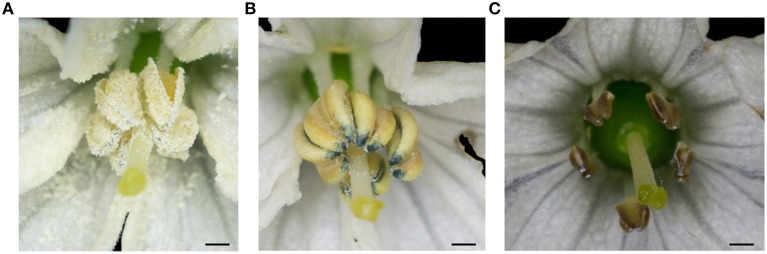
**The morphology of the anthers from the T_0_ transgenic plant (TCON8)**. **(A)** WT. **(B)** T_0_ transgenic plant (TCON8). **(C)** CMS lines HW203A. Bars = 1 mm.

Anthers of transgenic plants had few pollen grains that were not plump and either did not show dehiscence or did so only slightly (Figure 6A) compared to non-transgenic plants (Figure 6B). This indicated that the transgenic plants were semi-sterile, different from the CMS line (HW203A) whose shriveled anthers did not produce any pollen grains (Figure 6C).

To analyze the pollination ability, the transformants were used as male parent to pollinate the untransformed plants. The results showed that most of the pollinated flowers fell and nearly no fruit was set. Even though some small fruits were obtained, no seeds or very few seeds were observed (Table 2). These indicated that the pollen grains of the transformants were unable to pollinate.

**Table 2:** **Pollination test of T_0_ plants with non-transformed plants as female parent**.

**T_0_ plant**	**No. of pollinated flowers (WT♀ × T_0_♂)**	**Fruit-setting**	**No. of seeds (means ± *SE*)**
TCON3	15	2	1.50 ± 0.71
TCON5	15	0	0.00 ± 0.00
TCON8	15	0	0.00 ± 0.00
WT	15	8	58.42 ± 8.18

**Figure 7:**
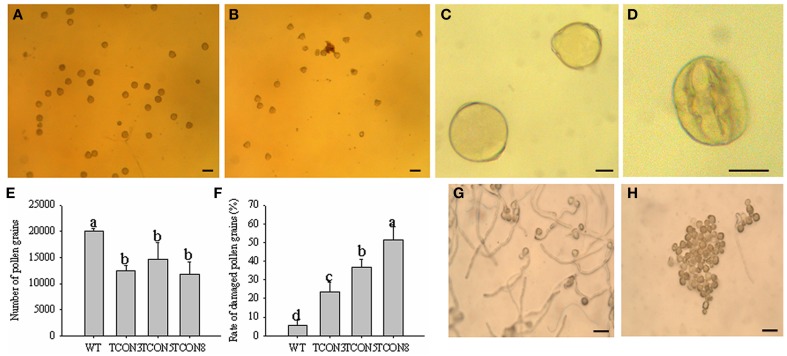
**Cytological analysis of the pollen grains in T_0_ transgenic plants**. **(A,C)** Pollen grains of WT. **(B,D)** Pollen grains of T_0_ transgenic plants. **(E)** Number of pollen grains in each anther. **(F)** Rate of damaged pollen grains. **(G)** Pollen germination of WT. **(H)** Pollen germination of T_0_ transgenic plants. **(A,B)** Bars = 100 μm; **(C,D)** Bars = 25 μm; **(G,H)** Bars = 200 μm.

The quantity of pollen grains in transgenic plants (T_0_) was significantly lower than in WT, but higher than in the CMS line (Figures 7A,B,E), which may have resulted from the slight dehiscence of the anthers in transgenic plants. Moreover, the rate of naturally damaged pollen grains in T_0_ (51.4%) (Figures 7D,F) was significantly higher than in WT (5.6%) (Figures 7C,F). Few germination of pollen grains was observed in T_0_ (Figure 7H), while nearly all pollen grains of WT produced normal pollen tubes (Figure 7G).

**Figure 8:**
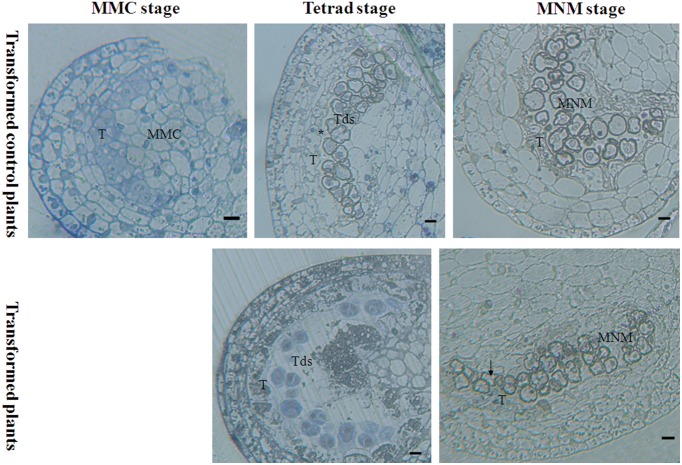
**Bright-field photographs of sections of anthers at different stages of development from TCON male sterile transformant and transformed control pepper plants**. At the MMC stage, anther development showed no differences in the transformant and control. T, tapetum; MMC, microspore mother cell; Tds, tetrad; MNM, mononuclear microspore; black arrow indicated the degraded mononuclear microspore; ^*^ nucleus of the tapetum cell. Bars = 25 μm.

Light microscopy that was used to examine the effect of *orf507* gene expression on the anther at different developmental stages revealed that at the mother microspore cell stage, the tapetal cell in the transformed plants showed no abnormalities compared to the transformed control plants, in which the tapetal cells were arranged closely with a dense cytoplasm (Figure 8). At the tetrad stage, tapetal cells of the transformed plants degraded, and no nucleus was observed, which is different from the CMS line in which the tapetum started to degrade but the cellular morph still existed until the mononuclear stage (Ji et al., 2013). In contrast, the tapetal cell of the transformed control plants developed normally and had many nuclei (Figure 8). Notably, at the mononuclear stage, the tapetum of the transformed control plants also degraded, but the anther chamber of the transformed plants became narrow, and the microspores lacked an integrated nucleus and were squeezed and distorted (Figure 8), compared to those of the transformed control plants (Figure 8). The results corroborated the analysis that showed the rate of natural damage to the pollen grains in transformed plants was significantly higher than in transformed control plants. All of these results demonstrated that expression of *orf507* driven by TA29 promoter led to premature PCD and then tapetal degeneration in anthers of transgenic plants, even earlier than in the CMS line (HW203A).

### Analysis of the BC_1_T_0_ generation of transgenic plants

The three transformants obtained were pollinated with untransformed plants. Germination tests of the BC_1_T_0_ seeds on filter paper containing kanamycin showed a 1:1 segregation ratio, which might indicated the presence of a single copy insert of the *orf507*, while no seeds of the untransformed plants germinated (Table 3).

**Table 3:**
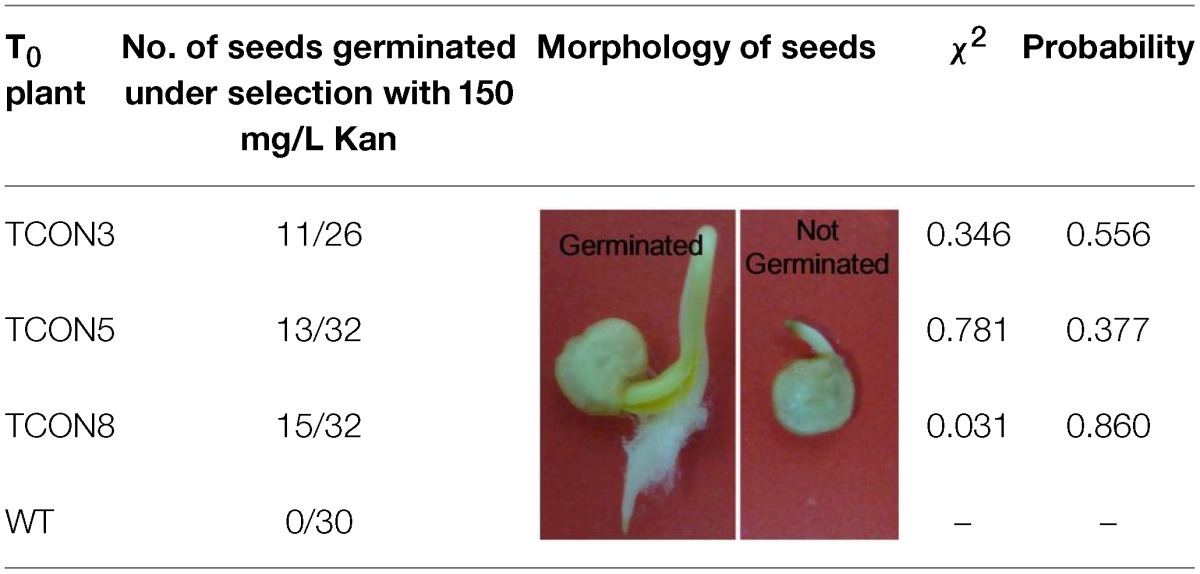
**Germination test of the BC_1_T_0_ seeds under selection with *Kanamycin***.

*Left of the slash: No. of seeds germinated under selection*.

*Right of the slash: No. of seeds used in the test*.

**Figure 9:**
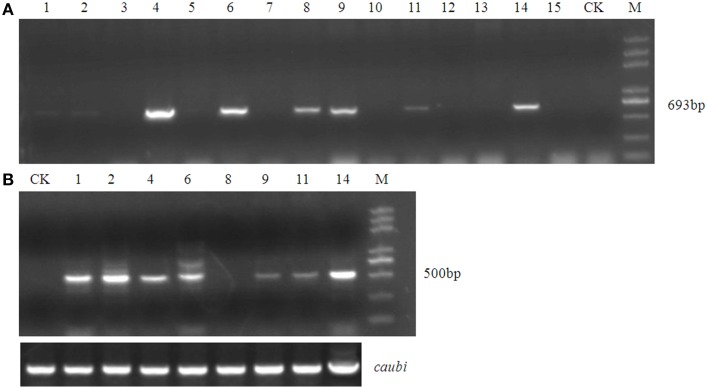
**Molecular analysis of test cross progeny of transgenic male sterile TCON 8 plant**. **(A)** PCR analysis using *Bam*HI *coxIV*F and *Sac*I *orf507*R primers. **(B)** RT-PCR analysis with *Kpn* I *orf507*F and *Sac*I *orf507*R primers. CK, transformed control plant; M, Trans2K plus marker; Lane1~15, progeny plant numbers; *caubi*, reference gene.

**Figure 10:**
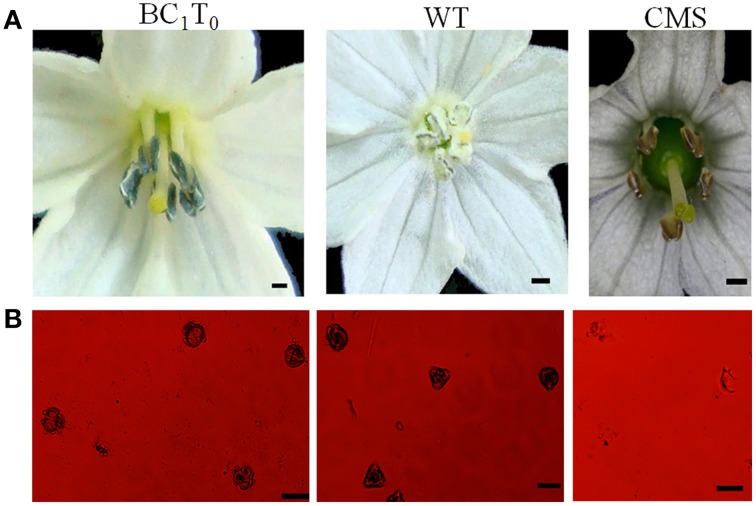
**Morphology of BC_1_T_0_ anthers (A) and pollen viability of BC_1_T_0_ plants (B)**. **(A)** Bars = 1 cm **(B)** Bars = 100 μm.

Fifteen plants were randomly selected from among the BC_1_T_0_ plants of TCON 8 and were grown without selection for PCR analysis. The results showed a 1:1 segregation for the transgene (Figure 9A), which might demonstrate the single copy insert of the transgene. However, this should be confirmed further by Southern blotting. RT-PCR for the presence of *orf507* transcripts in the anthers of PCR positive progeny plants confirmed the results of PCR analysis (Figure 9B). Because the obtained transgenic plants were semi-male sterile, and the degree of sterility differed significantly among individual plants, it was difficult to clearly evaluate co-segregation of the male sterility trait and the transgene, although about 80% of PCR positive plants in the progeny of TCON 8 were partially sterile. The morphology of the BC_1_T_0_ flowers with the transgene was similar to T_0_ plants (Figure 10A), while the anthers of the BC_1_T_0_flowers without the transgene were plump and produced plenty of pollen grains, which was similar to the WT plants. Above 95% of pollen grains of all progeny plants that were positive in the PCR analysis could not be stained, while the negative plants stained dark red (Figure 10B). No pollen grains were found in the CMS line. This indicated a certain co-segregation of the sterility trait with the transgene, albeit with incomplete penetrance in the BC_1_T_0_ progeny.

### Gene expression pattern in BC_1_T_0_ progeny

**Figure 11:**
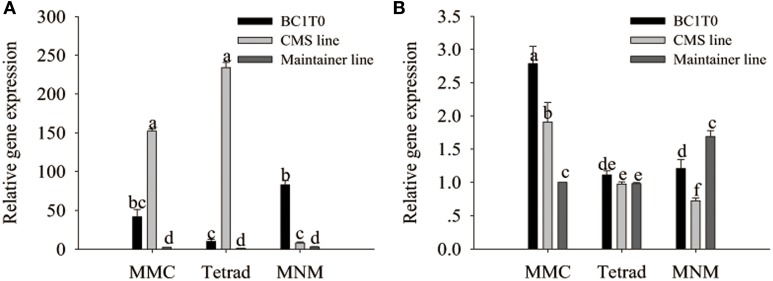
**qRT-PCR analysis of *orf507* (A) in buds of BC_1_T_0_, CMS line, Maintainer line, and *atp6-2*(B) in buds of BC_1_T_0_, Maintainer line (WT) and *ψ*atp6-2 (B) in CMS line**. Statistically significant differences between the means were determined using Fisher's LSD test (*p* < 0.05).

Previously, Li et al. (2013) confirmed the interaction of MtATP6 with ORF507 using the yeast two-hybrid analysis. Our investigation of the reaction of the *orf507* gene with the *atp6-2* gene using qRT-PCR showed that throughout all stages of development, the expression of the *orf507* gene in BC_1_T_0_ increased, while in the CMS line its expression gradually decreased, showing the changing trend with BC_1_T_0_, with no gene expression observed in the maintainer line. This might be due to the expression driven by different promoters in the CMS line and BC_1_T_0_ progeny (Figure 11A).

At the MMC and the MNM stages, we observed increased *orf507* gene expression and decreased *atp6-2* gene expression in BC_1_T_0_, which might indicate that expression of *orf507* inhibited expression of *atp6-2* to a certain degree (Figure 11B). Meanwhile, it might be confirmed by the up-regulated expression of the *atp6-2* gene in the maintainer line and the down-regulated expression in BC_1_T_0_. However, at the MMC stage, expression of *atp6-2* in BC_1_T_0_ was significantly higher than it was in the maintainer line, which might result from the regulation of other factors driven by the expression of *orf507* in BC_1_T_0_. The contradiction might be due to the regulation of other CMS-related genes on the *ψatp6-2* gene in the CMS line, or the mutation of the *ψatp6-2* gene affected the interaction of ORF507 with ATP6-2.

### Activity of mitochondrial enzymes in BC_1_T_0_ progeny

In the previous study (Ji et al., 2013), we have observed the dysfunction of the mitochondrial enzymes cytochrome *c* oxidase and the F_1_F_o_-ATP synthase in the CMS line of pepper plants. Meanwhile, histochemical localization of cytochrome *c* oxidase in the anther tapetum after the tetrad stage showed the opposite trend between the CMS line and the maintainer line. In addition, premature PCD of the tapetum in the transformants of TCON occurred at the tetrad stage. However, to date, we still do not understand whether the dysfunction of cytochrome *c* oxidase and the F_1_F_o_-ATP synthase in the CMS line was induced by expression of the *orf507* gene. To investigate whether the dysfunction was induced by expression of *orf507*, we analyzed the activity of the two enzymes in the anthers of BC_1_T_0_ progeny at the tetrad stage.

**Figure 12:**
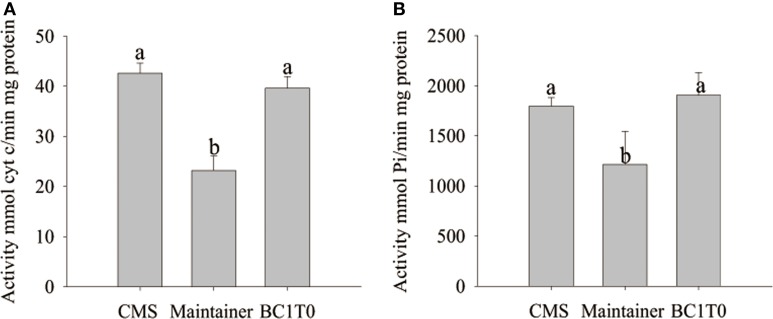
**Analysis of cytochrome *c* oxidase activity (A) and ATP hydrolysis activity of mitochondrial F_1_F_o_-ATP synthase (B) in buds of BC_1_T_0_, CMS line and Maintainer line (WT)**. Statistically significant differences between the means were determined using Fisher's LSD test (*p* < 0.05).

At the tetrad stage of development, activity of cytochrome *c* oxidase and the ATP hydrolysis of the F_1_F_o_-ATP synthase in BC_1_T_0_ progeny and the CMS line were not significantly different, and the levels in both types of plants were significantly higher than in the untransformants (maintainer line) (Figure 12). This indicated that expression of *orf507* in BC_1_T_0_ might lead to the increased activity of cytochrome *c* oxidase and ATP hydrolysis of the F_1_F_o_-ATP synthase at the tetrad stage, which was consistent with previous findings that suggested the enzyme activity in the CMS line was significantly higher than in the maintainer line at the tetrad stage (Ji et al., 2013). However, whether changes in ATP hydrolysis of the F_1_F_o_-ATP synthase were induced by the direct interaction of ORF507 on F_1_F_o_-ATP synthase or by a change in the activity of cytochrome *c* oxidase still needs to be confirmed by further studies.

## Discussion

CMS is known to be associated with novel *orf*s resulting from rearrangement of the mitochondrial genome (Schnable and Wise, 1998). In pepper, CMS is associated with the expression of a novel mitochondrial gene, *orf507*, which is expressed as a mitochondrial polypeptide in all the tissues of male-sterile plants (Kim et al., 2007; Gulyas et al., 2010; Ji et al., 2013). The CMS related *orf*s always regulate sterility by affecting the expression of the co-transcription gene, or by interacting with the protein and changing its biological function, such as the interaction of *orfH79* with the *atp6* gene in rice (Zhang et al., 2007). In pepper, *orf507* is co-transcribed with the *coxII* gene, and we also observed the dysfunction of mitochondrial cytochrome *c* oxidase in the CMS line. However, it remains unclear which tissue of the anthers undergoes the mutation that leads to the dysfunction of cytochrome *c* oxidase. In this study, by histochemial localization of cytochrome *c* oxidase, we found that differences between the CMS line and the maintainer line occurred mainly in the tapetum, through all the developmental stages of anthers, especially at the tetrad stage, which was similar to our previous study (Ji et al., 2013). Dysfunction of the mitochondrial enzyme is known to be associated with CMS (Zhang et al., 2007). As the marker enzyme, the aberrant cytochrome *c* oxidase will directly cause a change in the function of the mitochondria (Rich and Bonner, 1978). The tapetum provides nutrition and energy for the development of pollen grains (Pacini, 1997), so enzyme dysfunction in the tapetal cell might lead to a reduction of energy, which might in turn induce degradation of the tapetal cells. Then, premature PCD of the tapetum causes nutrient deficiency of the microspores, leading ultimately to male sterility.

Although the dysfunction of cytochrome *c* oxidase in the CMS line has been confirmed, we still do not understand the relationship of the CMS-specific *orf507* gene with cytochrome *c* oxidase and male sterility.

Cytochrome *c* oxidase is an enzyme complex that is being composed of 13 subunits and several metal prosthetic sites in mammals (Caar and Winge, 2003). Some CMS-related genes encode important subunits for or interact with the subunits of cytochrome *c* oxidase to affect its function. For example, in CMS-WA rice, the protein encoded by the CMS-specific WA352 gene interacts with COX11, which is encoded by a nuclear gene. In CMS-WA lines, WA352 accumulates in the anther tapetum, thereby inhibiting COX11 function in peroxide metabolism, triggering premature tapetum PCD, and consequent pollen abortion (Luo et al., 2013). For the co-transcription of the *orf507* gene with the *coxII* gene, we postulated that the ORF507 interacts with COXII which is firmly bound to subunit I and is the only subunit to possess a polar domain containing the binuclear Cu_A_ center. The Cu_A_ binuclear center is reduced by cytochrome *c* and then passed by an electron to cytochrome *a*, which in turn passes an electron to the cytochrome a_3_-Cu_B_ binuclear center and, finally, to the dioxygen molecule (Ostermeier and Lwata, 1996). The interference of COXII might cause the dysfunction of cytochrome *c* oxidase. The transgenic plants were generated using cotyledon of the maintainer line HW203B as explants. In addition, *orf507* was expressed under a constitutive expression promoter, CaMV35S, in the SCON vector. However, the elongated shoots infected with the SCON vector became etiolated and died gradually. We speculated that the toxic effect of ORF507 protein gradually accumulated on the shoot (Li et al., 2013), which inhibited development of the elongated shoots. Similarly, in CMS-HL rice, expression of *orfH79* leads to the excessive accumulation of ROS, a decrease in ATP synthesis, damage of the mitochondrial membrane potential and a decline in the rate of respiration, which finally inhibits root growth (Peng et al., 2010). The ORF507 protein might play a similar role, but empirical proof, such as the content of ROS, would be needed to provide evidence to support this hypothesis.

In the detection of the transformants using PCR and RT-PCR analysis, three transformants were confirmed to be positive for the presence of the *orf507* gene. Compared with the plump anthers of the maintainer line, the anthers of the transgenic plants were shriveled, barely dehisced, and could produce only few pollen grains; The quantity and germination rate of pollen was significantly lower in transformed plants than untransformed plants, while the natural damaged rate of pollen grains was higher in transgenic plants. Overall, this suggested that the transgenic plants were partially male-sterile. However, expression of the *orf456* gene in *Arabidopsis* induces complete male sterility (Kim et al., 2007). Male sterility in *Arabidopsis* might result from sequence alteration of *orf456*, or regulation of other CMS-related genes. In addition, expression of *orf507* was negatively correlated with the germination rate of pollen grains. Similar observations have been made in tobacco plants that were positive in the PCR analysis that were fertile and showed no expression or lower levels of expression of *orfH522* transcripts (Nizampatnam et al., 2009). It appears that expression of the CMS-related genes needed to exceed a threshold level to impair mitochondrial activity, and thereby induce male sterility. However, the toxic effect of ORF507 driven by the TA29 promoter was restricted to the tapetal cells, although the number of transgenic plants is insufficient to make the conclusion, which might prevent the accumulation of ORF507 from reaching the threshold level needed to trigger ablation of the tapetal cells (Smart et al., 1994). In the CMS line, the *orf507* gene is expressed in all tissues under the *coxII* promoter (Gulyas et al., 2010). The reason for the partial sterility of TCON transformants might include: (1) the CMS-related protein may not be localized in the appropriate compartments of mitochondria; (2) co-factors essential for the action of the CMS-related protein might not be found in heterologous systems; and (3) the protein might not be able to fold properly (Flavell, 1974; Duroc et al., 2006; Stockmeyer et al., 2007).

Expression of the *orf507* gene in HW203B affected only the development of the tapetum, and not other tissues of the anthers or their maturation. Ablation of the tapetum occurred in a pattern that is typical of the reported TA29 expression profile (Koltunow et al., 1990). At the tetrad stage the tapetal cells in the transgenic plants almost completely degraded. At the MNM stage, though transgenic plants could produce microspores, the microspores were shrunken, distorted and damaged. After flowering, pollen grains collected from the anthers were also damaged.

As the anthers developed, the trend for the expression of the *orf507* gene in BC_1_T_0_ was contrary to what was found in the CMS line, which might be due to the expression driven by different promoters in the CMS line and BC_1_T_0_ progeny. The interaction of ORF507 with MtATP6 reduced the activity of the mitochondrial F_1_F_o_-ATP synthase and the ATP content (Li et al., 2013). In this study, expression patterns of *orf507* and *atp6-2* in BC_1_T_0_ progeny were opposite. Moreover, the opposing trends for the two genes in BC_1_T_0_ were different from what was seen in the CMS line. These findings might suggest that there were other CMS-related genes in the CMS line that interacted with the ATP6-2 subunit, or that the mutation of *ψatp6-2* altered the interaction with ORF507.

We still do not understand whether dysfunction of cytochrome *c* oxidase and the F_1_F_o_-ATP synthase in the CMS line was induced by the expression of *orf507*. Cytochrome *c* oxidase (EC 1.9.3.1) plays a crucial role in aerobic life due to its specific capability to accept an electron from cytochrome *c* and then transfer it to a dioxygen molecule to form water, while pumping protons across the mitochondrial membrane to produce 2.5 ATP (Wikström, 2000). The abnormal increase in the activity of cytochrome *c* oxidase induced by *orf507* expression, causing an increased ability to transfer electrons, would disorder the transfer of electrons, and thus destroy the membrane potential (Pecina et al., 2004). Altering the pH of the cell and disrupting the electrochemical gradient may cause ATP synthase to hydrolyze ATP rather than synthesis ATP (Cabezón et al., 2000; García-Trejo and Morales-Ríos, 2008). With ATP hydrolysis, protons are transported from the matrix to the inter-membrane space to rectify the membrane potential (Crompton, 1999). Expression of *orf507* increased the hydrolysis of ATP of F_1_F_o_-ATP synthase, which resulted in premature ATP hydrolysis. Concurrently, the imbalance between ATP synthesis and consumption would aggravate the sustainable accumulation of ROS and lead to oxidative stress beyond the regulation of the antioxygen system in mitochondria. This would subsequently destroy the structure and the active site of the mitochondrial enzyme, thus disrupting the enzyme activity. ATP is an important determinant of the integrity of mitochondria. Lack of ATP will limit vegetative growth, therefore the CMS plants were ATP deficient (Teixeira et al., 2005). The imbalance between energy expenditure and production exacerbates the defects of the proteins in the respiratory chain complexes, and further deteriorates the state of mitochondrial disorder (Wan et al., 2007). These changes could lead to the premature PCD and ablation of plant cells that we observed.

Mitochondrion is a semi-self-organelle in plant cells. Unlike nuclear DNA, which has no protective histones combined with mtDNA, thus it is easily impaired by excessive ROS (Singh, 2004). Therefore, a test of mtDNA fragmentation would further ascertain the relationship between the *orf507* gene and excessive ROS accumulation.

## Conclusions

This report provides direct evidence that dysfunction of cytochrome *c* oxidase in the tapetum might be responsible for male sterility in pepper and the *orf507* gene might be one of the causative factors of male sterility in pepper CMS line, building on the indirect evidences accumulated by several earlier reports. We have also established that *orf507* alone is insufficient to induce complete male sterility in a heterologous system of pepper, which was different from *orf456* inducing complete male sterility when transformed into *A. thaliana*. In future, discovery of more CMS-related genes and elucidation of their functions will help to unravel the mechanism of complete male sterility in pepper.

## Author contributions

JJ, ZL, and ZG conceived the research. JJ, WH, YY, and ZL performed the research. JJ, WH, YL, DL performed interpretation of data. JJ, ZL, and DL took the photos. JJ, YY, and WC performed statistical analyses. JJ and ZG wrote the paper. JJ, DL, and YY revised the paper. ZG and WC provided the materials and resources for the research. JJ, WH, WC, and ZG performed the integrity of the work. All authors read and approved the final manuscript.

### Conflict of interest statement

The authors declare that the research was conducted in the absence of any commercial or financial relationships that could be construed as a potential conflict of interest.

## Supplementary material

Supplementary Figure 1 **Generation of the transformed plants by means of leaf disc method**. **(A)** Bud differentiation. **(B)** Shoot elongation. **(C,D)** Rooting stage, Rooting plant. **(E)** Transplanted plant.
