# Supplementary figures and images for: Tapetum-specific expression of a cytoplasmic orf507 gene causes semi-male sterility in transgenic peppers
Source: Front Plant Sci. 2015 Apr 22;6:272. doi: 10.3389/fpls.2015.00272 (PMC4406146; doi:10.3389/fpls.2015.00272)

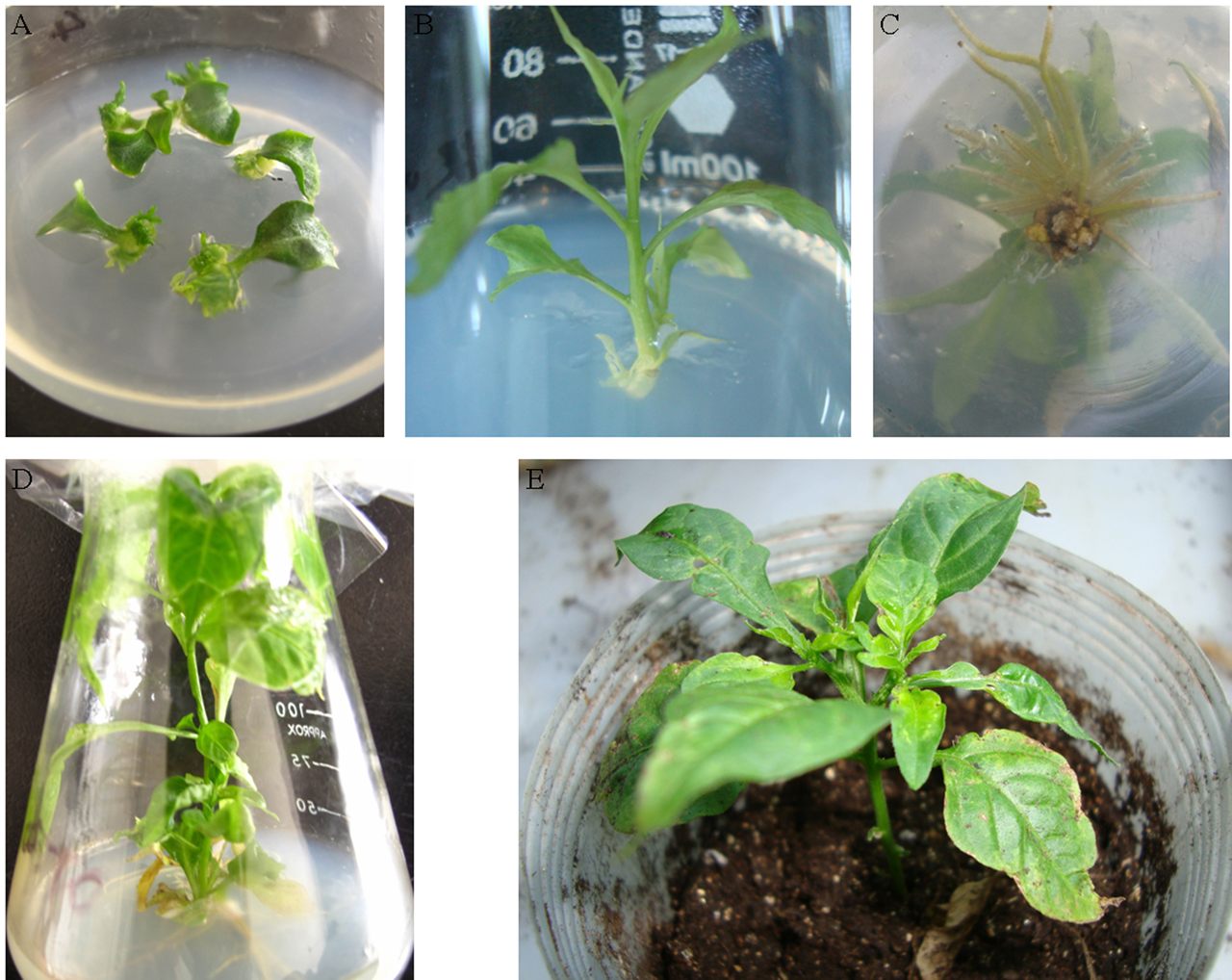

Supplement: Supplementary Figure 1 — Generation of the transformed plants by means of leaf disc method. (A) Bud differentiation. (B) Shoot elongation. (C,D) Rooting stage, Rooting plant. (E) Transplanted plant. [file Image1.TIF]
